# Epidemiology of acute myeloid leukemia in Virginia: Excellent survival outcomes for patients in rural Appalachia

**DOI:** 10.1002/cnr2.1354

**Published:** 2021-03-09

**Authors:** Krista M. Isaac, Daniel R. Reed, Raj Piyush Desai, Eli Williams, Rajesh Balkrishnan, Michael K. Keng, Karen K. Ballen

**Affiliations:** ^1^ Division of Hematology/Oncology University of Virginia Health System Charlottesville Virginia USA; ^2^ Section on Hematology/Oncology Wake Forest Baptist Comprehensive Cancer Center Winston‐Salem North Carolina USA; ^3^ Cancer Population Health Core University of Virginia Cancer Center, University of Virginia School of Medicine Charlottesville Virginia USA; ^4^ Department of Pathology University of Virginia Health System Charlottesville Virginia USA

**Keywords:** AML, cytogenetics, geography, socioeconomic factors, telemedicine

## Abstract

**Background:**

Acute myeloid leukemia, the most common acute leukemia in adults, has a poor overall survival. Studies have suggested that certain socioeconomic factors such as living in a rural or farming area are associated with worse outcomes. Since 42% of acute myeloid leukemia patients seen in our academic center reside in a rural area, we have a unique opportunity to study outcomes of patients in rural versus urban settings.

**Aim:**

This analysis evaluates the effect of geography and socioeconomic factors on the biology, treatment, and overall survival of patients with acute myeloid leukemia, with the goal of understanding health care disparities.

**Methods and results:**

Patient characteristics, cytogenetic data, treatment history, and overall survival were collected and analyzed to identify differences between urban and rural residency. This cohort included 42% of patients who resided in a rural area at the time of acute myeloid leukemia diagnosis. There was no difference in overall survival between the cohorts. The 1 year overall survival for the entire cohort was 47.9%. There was no difference detected in rates of adverse cytogenetics between the rural and urban cohorts. Similar numbers of patients received induction chemotherapy or proceeded to allogeneic stem cell transplant between the cohorts.

**Conclusions:**

This study highlights that similar outcomes can be achieved in rural and urban patients, suggesting that intensive efforts at telehealth, education, and collaboration with local oncology practices may be beneficial.

## 1 BACKGROUND

Acute myeloid leukemia (AML) is the most common acute leukemia in adults. The incidence of AML is currently 1.2% per year, with a disappointing 5‐year overall survival (OS) of 28.3%.^1^ Currently, the effect of area of residency, specifically living in a rural area as compared to an urban area, on the mutational frequency and OS in AML is unknown. According to the 2010 census, 24.5% of Virginia's population currently lives in a rural area.^2^ Our cancer center provides care to a significant number of patients residing in rural communities, including many from rural Appalachia. This provides a unique opportunity to study health care disparities in our state, and the effects of geography and socioeconomic factors on the genetics and outcomes of patients with AML.

Cytogenetic analyses and molecular genetic testing have prognostic importance in AML. The 2017 European Leukemia Net (ELN) criteria are the most widely used risk model and stratifies patients into three prognostic groups: favorable, intermediate, and adverse.^3^ Unfortunately, there are many gene mutations which are not currently characterized in this model that may have a significant impact on prognosis such as *NRAS*/*KRAS* or *DNMT3a* mutations.^4^, ^5^, ^6^ Recent advances using targeted therapy in patients who are not candidates for intensive induction chemotherapy and who have relapsed or refractory disease have shown response rates of 20–60%.^1^, ^7^, ^8^, ^9^, ^10^, ^11^

Despite the progress made in cancer detection, treatment, and survival, patients from rural Appalachia continue to have higher incidence of cancer diagnosis and cancer‐related mortality compared to patients from non‐Appalachia.^12^, ^13^, ^14^ Although the gap in incidence of cancer has narrowed, patients from Appalachia continue to have increased rates of tobacco‐related cancers, specifically lung cancer, oral cavity cancer, pharyngeal cancer and cancer of larynx.^15^ In Virginia, the cancer mortality rate in Appalachia is 10% higher than the national rate and 11% higher than non‐Appalachian Virginia.^16^ Our Cancer Center provides strategic services to rural residents including those from Appalachia and throughout Virginia to address disparities in cancer mortality. This rural cancer program benefits from extensive local, regional, and state‐wide partnerships with regional outreach staff to support community‐based participation and bi‐directional communication between rural residents, communities and the academic cancer center. A representative Community Advisory Committee advises on strategic partnerships with health systems, rural care providers, churches, and community organizations to deliver culturally adapted, evidence‐based programming, intervention, and screening services.

The etiology of the increased incidence of cancers in rural Appalachia is likely multifactorial and related to health care disparities and environmental factors. Certain environmental exposures, such as living in a rural or farming community, have been associated with an increased risk for the development of AML.^17^ The Iowa Women's Study found that women who lived on a farm or in a rural area were twice as likely to develop AML as compared with women who lived in a city (relative risk 2.38).^18^ Further analysis demonstrated that even women who did not identify as a farmer but who lived on farms or in rural communities still had an increased risk of the development of AML.^19^

The effect of geographic residence on genetic characteristics of AML is poorly understood. In addition, the impact of geographic residence on molecular characteristics is not well studied. In a study evaluating a cohort of AML patients from Florida and Arizona, several lifestyle and environmental factors, such as rural or farm habitats, obesity, and smoking, were evaluated to determine their effect on cytogenetics and outcomes of AML.^17^ Although only 5% (*n* = 14) of the cohort resided in a farm habitat, more than half of those patients had poor risk cytogenetics. Farm residence was not found to be a statistically significant predictor of poor risk cytogenetics (odds ratio: 2.65, *p* value: .087).^17^ Molecular characteristics were not included in this study.^17^

Other patient characteristics such as race or ethnicity have also been demonstrated to impact outcomes. A retrospective analysis of 2570 patients with de novo AML found that African American patients (10.5% of the study population) presented at a younger age and were more likely to have favorable risk cytogenetics. Despite these good prognostic factors, African American men had lower complete remission (CR) rates as compared with African American women or white patients.^20^ The authors of this study surmised the lower CR rates were due to biologic factors as there were no differences in other prognostic markers, induction regimens, or treatment‐related toxicities.^20^

Additionally, poverty has been associated with worse outcomes. A study evaluating the impact of non‐biological factors on AML outcomes demonstrated that patients who were within the lower three quintiles of median household income had worse OS.^21^ In an analysis of patients with AML, acute lymphoblastic leukemia (ALL), and myelodysplastic syndrome (MDS), patients with higher levels of poverty were less likely to undergo allogeneic hematopoietic cell transplantation (HCT).^22^ In this study, there was no difference in HCT based on rural or urban residency.^22^

In our study, the molecular characteristics of patients with AML in rural and urban Virginia were compared. The effect of socioeconomic factors, including geographic residency, on AML biology and outcomes was also investigated.

## 2 METHODS

We performed a single center retrospective study of AML patients diagnosed between 9/2015 and 12/2019. Consecutive patients with a new diagnosis of AML and a clinical next generation sequencing panel (Illumina TruSight Myeloid 54 gene panel) performed during their initial leukemia workup were identified and included. Patients were excluded if they had a diagnosis of acute promyelocytic leukemia or chronic myeloid leukemia in blast crisis. Patients were also excluded if Trusight Myeloid Panel was not obtained at initial diagnosis.

Data collected included sex, date of birth, state of residency, zip code, date of AML diagnosis, smoking history, height, weight, prior history of MDS or myeloproliferative neoplasm (MPN), cytogenetic data, treatment regimen (including HCT), date of treatment, number of therapies, remission date, relapse date, last follow up date, and date of death. Racial and ethnic data were collected based on self‐reported data that was documented in the electronic medical record.

The zip codes of the patients were used to identify the county in Virginia they reside in using the U.S. Department of Housing and Urban Development United States Postal Service Zip Code Crosswalk data. For zip codes that belong to more than one county, the characteristics of the dominant county (based on population density) were used. Rural‐Urban Commuting Area codes were used to identify the urban‐rural residential areas based on zip codes as previously described.^23^, ^24^, ^25^ Geographic‐level socioeconomic data were extrapolated from the individual counties based on the Area Health Resource Files (AHRF) database and used to determine percent ratio of income to poverty and percent high school or above education. The distance from the cancer center to the zip code of the residence of the patients was calculated using the SAS procedure “zipcitydistance.” This program calculates the geodetic distance in miles between two zip code locations. The centroid of each zip code is used in the calculation.

Molecular and genetic data were collected and patients were assigned either favorable, intermediate or poor prognosis risk groups according to published guidelines.^3^ Mutational profile, cytogenetics, and OS were compared between the groups. Mutations analyzed were limited to *ASXL1*, *DNMT3A*, *FLT3* (internal tandem duplication and tyrosine kinase domain), *IDH1*, *IDH2*, *NPM1*, *NRAS*, *TET2*, and *TP53*, as these are often used in medical decision making.

The treatment regimen was recorded as induction chemotherapy if the patient was treated with an anthracycline and cytarabine; liposomal daunorubicin and cytarabine; mitoxantrone, etoposide and cytarabine; etoposide and cytarabine; or on a clinical trial of induction‐based chemotherapy. The treatment regimen was recorded as non‐induction if the patient was treated with low dose cytarabine, a hypomethylating agent (azacitidine or decitabine), low dose cytarabine and venetoclax; a hypomethylating agent and venetoclax, gemtuzumab ozogamicin, supportive care only or on a clinical trial of non‐induction chemotherapy.

## 3 STATISTICAL ANALYSIS

Bivariate associations were calculated to determine if each variable in this study was associated with either the groups (urban or rural area of residency). Time to event data were analyzed using log‐rank tests, Kaplan–Meier method, and Cox proportional hazard regression model. The OS was estimated using Kaplan–Meier product limit by area of residency, treatment type, and transplant. Multivariable analyses based on Cox Proportional Hazards regression model were used to examine the effect of the various covariates on the risk of dying by area of residence. Statistical tests were two‐sided at .05 significance level. All analyses were performed using SAS version 9.4.

## 4 RESULTS

### 4.1 Patient characteristics

**FIGURE 1:**
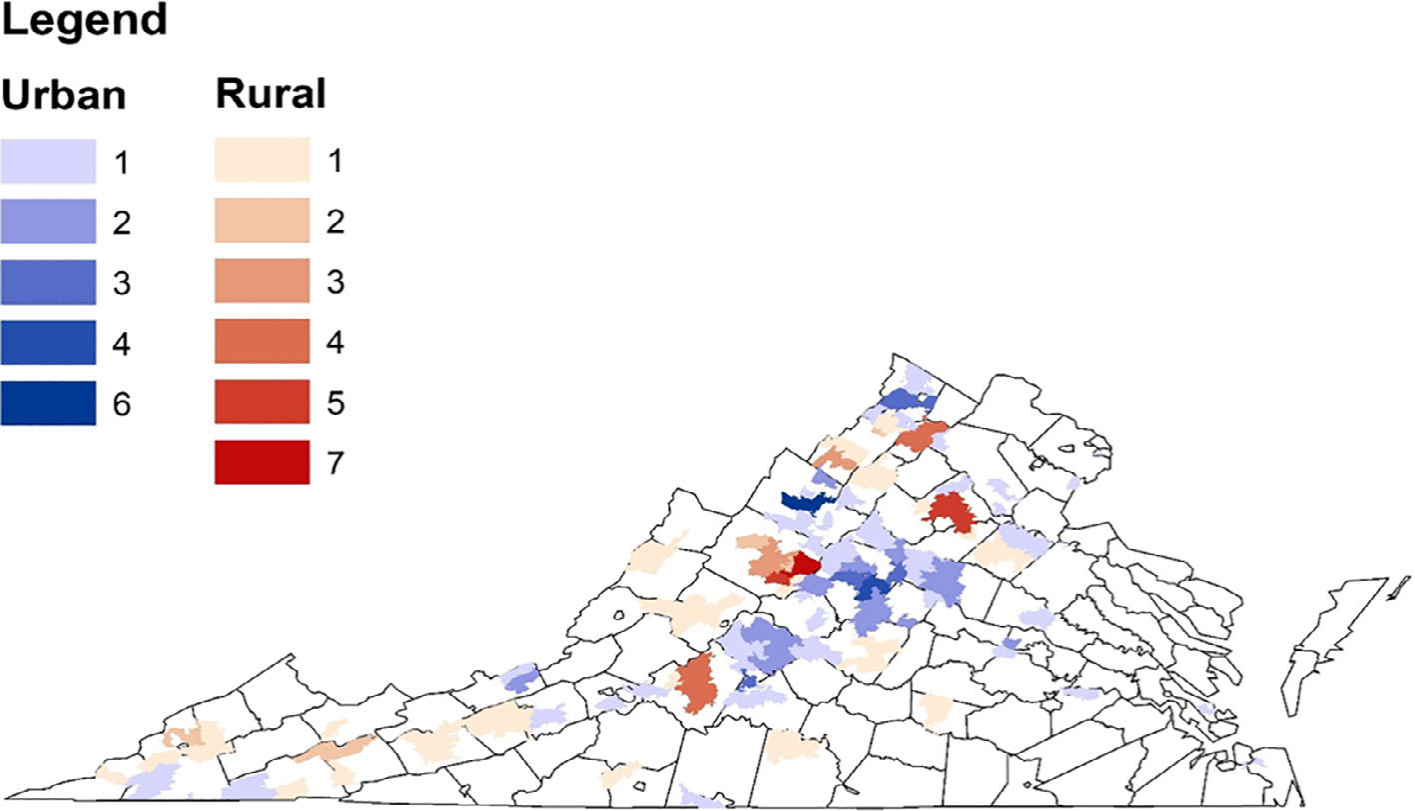
Map of frequency of patients per zip code. This figure displays the frequency of patients per zip code. Rural patients are denoted in red; urban patients are denoted in blue

A total of 163 patients were identified who met criteria for inclusion in this study. Table 1 demonstrates the patient characteristics stratified by area of residence. Median age of the entire cohort was 65.4 years old. 58.9% (*n* = 96) were male. 41.7% (*n* = 68) of patients were from a rural area; 58.3% (*n* = 95) were from an urban area. 15.34% (*n* = 25) of patients were from a county in Appalachia; 33.33% (*n* = 13) were from rural Appalachia; 9.68% (*n* = 12) were from urban Appalachia. A map of the area of residency stratified by rural versus urban is demonstrated in Figure 1. Patients from a rural area were more likely to live below the poverty level as compared to the urban cohort (13.55 vs. 11.57%, *p* = .0304). Patients from a rural area were less likely to have graduated high school as compared to the urban cohort (85.57 vs. 87.79%, *p* = .004). There was no difference in median age, race, prior MDS or MPN, number of patients who underwent chemotherapy or number of patients who underwent HCT between the rural and urban cohorts, as demonstrated in Table 1.

**TABLE 1:** Patient characteristics

Characteristics	Urban (*n* = 95)	Rural (*n* = 68)	*p* Value
Age	64.96	66.65	.7673
Sex			
Male	53 (55.8%)	43 (63.2%)	.3407
Female	42 (44.2%)	25 (36.8%)	
Race			
Non‐Hispanic White	84 (88.4%)	61 (89.7%)	.7964
Others	11 (11.6%)	7 (10.3%)	
Smoking Status			
Current Smoker	13 (13.7%)	14 (20.6%)	.3585
Former Smoker	35 (36.8%)	18 (26.5%)	
Never Smoker	47 (49.5%)	36 (52.9%)	
Risk Category (ELN classification)			
Favorable	13 (13.7%)	4 (5.9%)	.2224
Intermediate	39 (41.1%)	27 (39.7%)	
Adverse	43 (45.3%)	37 (54.4%)	
Prior MDS or MPN	20 (21.1%)	15 (22.1%)	.8774
Chemotherapy			
Induction	67 (70.5%)	41 (60.3%)	.1731
Non‐induction	28 (29.5%)	27 (39.7%)	
HCT	23 (24.2%)	17 (25%)	.9081
Mean percent below poverty level	11.57%	13.55%	**.0304**
Mean percent high‐school graduate	87.79%	85.57%	**.0004**
Distance traveled (in miles)	58.05	80.31	**.0290**

*Note*: This table displays the patient characteristics stratified by area of residency (urban or rural). There was no statistical difference between age, sex, race, smoking status, risk category, prior MDS or MPN, or treatment regimen (including HCT). There was a statistically significant difference between the cohorts for the percentage of patients living below the poverty level and the percentage that was high‐school graduates (*p* values of .0304 and .0004, respectively). There was also a statistically significant difference in the distance traveled between the cohorts (*p* value .029).

Abbreviations: ELN, European Leukemia‐Net; HCT, allogeneic stem cell transplant; HR, hazard ratio; MDS, myelodysplastic syndrome; MPN, myeloproliferative neoplasm.

### 4.2 Cytogenetics and molecular characteristics

Patients from both cohorts had similar risk cytogenetics (urban cohort: favorable: *n* = 13, 13.9%; intermediate: *n* = 39; 41.1%; adverse: *n* = 43; 45.3%; rural cohort: favorable: *n* = 4, 5.9%; intermediate: *n* = 27; 39.7%; adverse: *n* = 37, 54.4%; *p* = .22). Molecular data were available for all patients at time of diagnosis. There were similar frequencies of all the mutations analyzed in both the urban and rural cohorts (Table 2). The most frequently identified mutations were DNMT3a (urban cohort: *n* = 19, 20%; rural cohort: *n* = 9, 13.2%) and NPM1 (urban cohort: *n* = 18, 19%; rural cohort: *n* = 9, 13.2%). There was similar frequency in ASXL‐1, a poor prognosis mutation, in both cohorts: ASXL‐1 was identified in 2% (*n* = 2) and 5.9% (*n* = 4) of the urban and rural cohorts, respectively.

**TABLE 2:** Mutation frequency by area of geographic residency

	Urban	Rural	*p* Value
	*n* (%)	*n* (%)	
*ASXL1*	2 (2%)	4 (5.9%)	.2362
*DNMT3a*	19 (20%)	9 (13.2%)	.2589
*IDH1*	5 (5.3%)	4 (5.9%)	1.0000
*IDH2*	6 (6.3%)	2 (2.9%)	.4705
*NPM1*	18 (19%)	9 (13.2%)	.3334
*NRAS*	7 (7.4%)	4 (5.9%)	.7634
*TET2*	3 (3.2%)	1 (1.5%)	.6411
*TP53*	4 (4.2%)	5 (7.4%)	.4924
*FLT3*	8 (8.4%)	7 (10.3%)	.6833

*Note*: This table describes the mutation frequency stratified by area of residency (urban or rural). There was no statistically significant difference in the frequency of any mutations between the cohorts. *FLT3* incorporates both internal tandem duplication and tyrosine kinase domain mutations.

### 4.3 Survival outcomes

**FIGURE 2:**
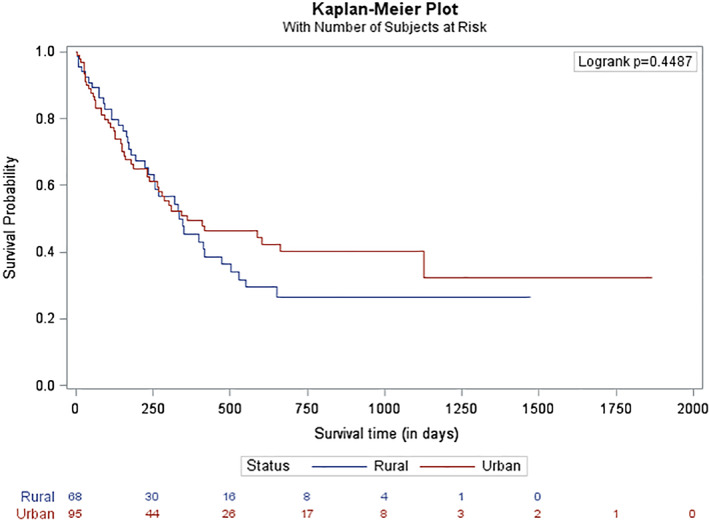
Overall survival by area of geographic residency. This figure demonstrates the overall survival stratified by area of geographic residency

**FIGURE 3:**
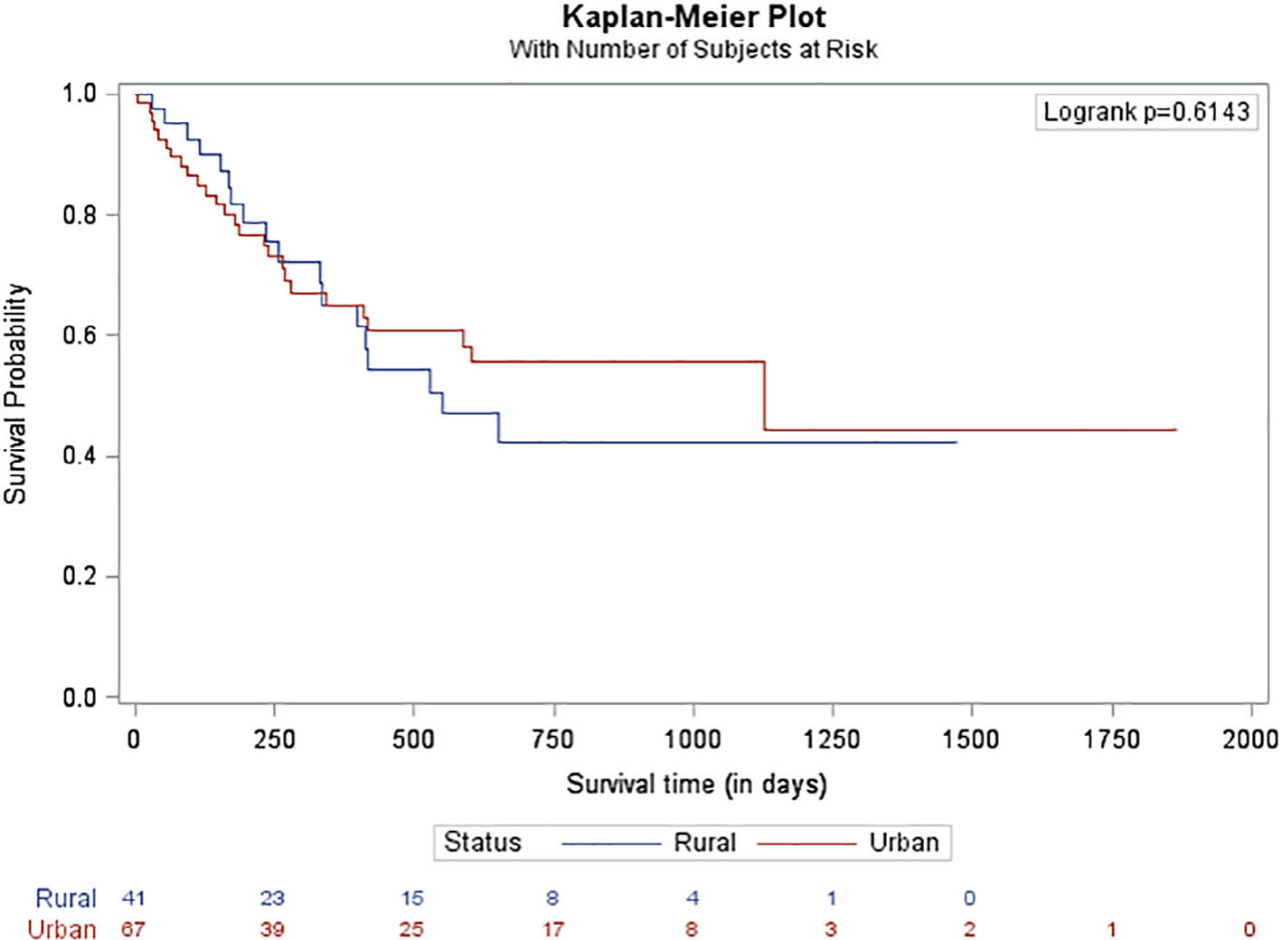
Overall survival of patients undergoing induction chemotherapy. This figure demonstrates the overall survival for patients who underwent induction chemotherapy (defined as receiving an anthracycline and cytarabine; liposomal daunorubicin and cytarabine; mitoxantrone, etoposide and cytarabine; etoposide and cytarabine; or treated on a clinical trial of induction‐based chemotherapy) stratified by area of geographic residency

The 1 and 2 year OS rate for the entire cohort was 47.9 and 29.6%, respectively. The 1 and 2 year OS rate for the rural cohort was 45.4 and 26.55% (*n* = 68) and for the urban cohort was 49.3 and 32.33% (*n* = 95; *p* = .4487; Figure 2). The median follow‐up for the entire cohort was 230 days; 137 patients have follow‐up of 2 years or less. A total of 108 patients (66.3%; urban: *n* = 67, 70.5%; rural: *n* = 41, 60.3%; *p* = .1731) underwent induction chemotherapy. There was no difference in survival for patients who underwent induction chemotherapy when stratified by rural and urban cohorts (42.3 vs. 44.5 months, *p* = .6143, Figure 3). The 1 and 2 year OS for patients who underwent induction chemotherapy was 65.2 and 50.4%. Forty patients underwent an HCT: 23 patients (24.2%) in the urban cohort and 17 patients (25%) in the rural cohort. The 1 and 2 year OS for patients who underwent HCT was 83.4 and 76.6%. There was no difference in survival for patients who underwent HCT when comparing the rural and urban cohorts (63.5 vs. 86.0 months, *p* = .1792, Figure 4).

**FIGURE 4:**
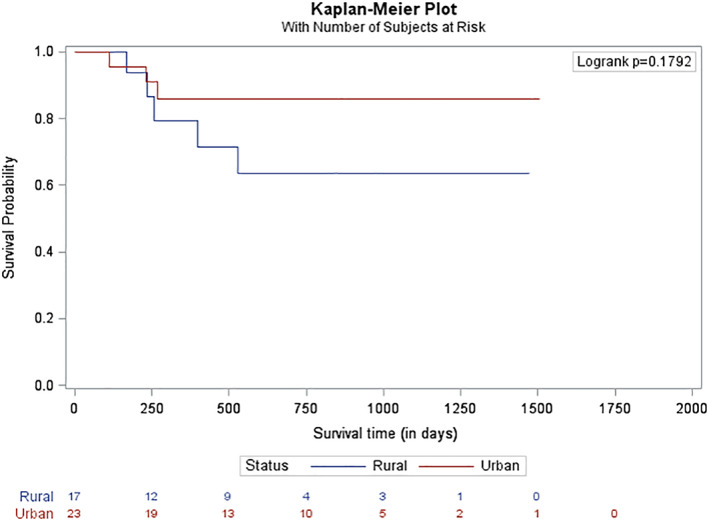
Overall survival of patients undergoing allogeneic stem cell transplant. This figure demonstrates the overall survival for patients receiving an allogeneic stem cell transplant stratified by area of residency

### 4.4 Socioeconomic characteristics

Data on the median income and high school graduation rate was available at the county level. In the rural cohort, 13.5% of patients lived in a county with a median income below the poverty level; in the urban cohort, 11.6% of patients lived in a county with a median income below the poverty level (*p* = .03). The rate of high school graduation from county data was lower in the rural cohort as compared to the urban cohort (85.6 vs. 87.8%, *p* = .0004). Rural patients traveled an average of 80.3 miles for treatment; urban patients traveled 58.05 miles (*p* = .029).

### 4.5 Multivariate analysis

Multivariate analysis (Table 3) demonstrates that receiving an allogeneic stem cell transplant was associated with increased survival (HR 0.140, CI 0.058–0.336, *p* = <.0001). In addition, when compared to patients with adverse cytogenetics, patients with favorable or intermediate risk cytogenetics had improved survival (HR 0.278, CI 0.104–0.740, *p* = .010; HR 0.398, CI 0.222–0.715, *p* = .002). Residency in a rural area was not associated with decreased survival as compared to an urban area (HR 1.229, CI 0.716–2.108, *p* = .455). Most patients in this study identified as non‐Hispanic White (urban cohort: *n* = 84, 88.4%; rural cohort: *n* = 61, 89.7%); race was not predictive of survival (HR 1.396, CI 0.649–3.002, *p* = .394). Poverty, as determined by mean percentage of the county living at or below the poverty level, was not predictive of survival (HR 0.987, CI 0.941–1.036, *p* = .602). Percentage of patients in the county with a minimum of a high school education was also not predictive of survival (1.069, CI 0.979–1.166, *p* = .136).

**TABLE 3:** Impact of patient characteristics on survival

Characteristic		Hazard ratio	95% HR CL		*p* Value
Residency	Rural vs. Urban	1.229	0.716	2.108	.455
Age		1.020	0.999	1.042	.068
Sex	Female vs. Male	1.067	0.583	1.953	.833
Race	Others vs. White	1.396	0.649	3.002	.394
BMI category	Obese vs. Normal	1.034	0.549	1.945	.918
BMI category	Overweight vs. Normal	0.626	0.327	1.198	.157
BMI category	Underweight vs. Normal	0.786	0.355	1.739	.552
Risk category	Intermediate vs. Adverse	0.398	0.222	0.715	**.002**
Risk category	Favorable vs. Adverse	0.278	0.104	0.740	**.010**
Prior MDS or MPN	No vs. Yes	0.666	0.347	1.275	0.220
HCT	Yes vs. No	0.140	0.058	0.336	**<.0001**
Ratio of income to poverty		0.987	0.941	1.036	0.602
Percent with minimum high school education		1.069	0.979	1.166	0.136
Distance traveled, in miles		1.003	0.998	1.008	0.217

*Note*: This table displays the cox regression analysis for this study. Factors that were predictive for OS including age, HCT, and risk category. The remaining factors were not predictive for OS.

Abbreviations: BMI, body mass index; HCT, allogeneic stem cell transplant; HR, hazard ratio; MDS, myelodysplastic syndrome; MPN, myeloproliferative neoplasm. Bold values are statistically significant.

## 5 DISCUSSION

To our knowledge, this is the first report comparing AML mutations and outcomes for rural versus urban patients, including 15% from counties in Appalachia. Studies of outcomes of other cancers for patients from Appalachia suggest outcomes are worse compared to non‐Appalachia patients.^12^, ^13^, ^14^, ^26^ Our retrospective study did not demonstrate inferior outcomes for rural patients. Our study included 42% of patients from a rural area of whom 33.33% live within rural Appalachia. No differences were seen between the urban and rural cohorts, specifically in age, sex, race, and smoking status, history of prior MPN or MDS or treatment with transplant. A main hypothesis prior to initiating this study was that there would be a biologic difference between the cohorts; there was no statistical difference in cytogenetic and genetic abnormalities tested, suggesting the biology of the disease is similar between rural and urban cohorts.

Studies have suggested multiple factors for the disparities observed in rural communities including comorbid conditions lower socioeconomic status, low rates of health insurance, and greater distances to obtain health care.^12^, ^27^, ^28^ In a descriptive survey design study, Huttlinger and colleagues reported that patients from Southwest Virginia had a higher level of chronic diseases (hypertension, obesity, diabetes) than the rest of Virginia.^27^ We did not collect information on chronic diseases other than obesity, which did not predict for survival. The rural patients treated at the cancer center could have lower rates of comorbidities as compared to that described by Huttlinger and colleagues which may be one factor to explain their better outcomes. Future studies on AML outcomes from our center should include comorbidities to evaluate whether this contributes to the similar outcomes between rural and urban cohorts.

Lower socioeconomic status also contributes to cancer care disparities.^26^, ^29^ Rural patients in our study had a lower education level and were more likely to live below the poverty level. However, it is important to recognize that socioeconomic status from our study was extrapolated from county data and does not reflect the individual patient socioeconomic status. We did not have access to individual patient education level, occupation or income level. Further studies will include these factors in addition to race to more completely assess the influence of social determinants of health.^30^, ^31^

Traveling a distance to receive care has been associated with worse outcomes. Rotz et al found living distances greater than 50 miles from a treatment center was associated with inferior outcomes in patients with acute lymphoblastic leukemia but not in patients with AML.^32^ Similarly, our study did not demonstrate inferior outcomes with a greater distance traveled for treatment. On average, rural patients in our study traveled 80.3 miles compared to 58.05 miles for urban patients. Although this difference in distance traveled is statistically significant, there was no impact on OS. This may be attributable to the inpatient treatment of AML: patients require admission for induction chemotherapy thus decreasing the burden of traveling on the patient and ensuring patients receive the entire course of treatment.

Despite the difference in distance traveled to receive care at our center, there was no difference in the frequency of patients proceeding to HCT. HCT is potentially curative therapy for high risk AML, and based on adverse cytogenetics in our population, an estimated 80 patients might have benefited from transplant. Only 40 patients in our cohort received HCT. Other work from our center suggests that less than 50% of patients eligible for HCT receive an HCT in the state of Virginia, and access is particularly poor for patients that reside in areas with a high African‐American population or for patients with government‐based insurance.^33^ Data on these factors were not collected in this analysis; further studies are needed to better elucidate this finding.

AML offers unique challenges to the outpatient management of patients after discharge from a tertiary care center. Unlike breast cancer and other solid tumor patients that are typically treated in the outpatient, community setting, acute leukemia patients require frequent laboratory checks with the resources to support frequent blood and platelet transfusions. Acute leukemia patients can become severely ill quickly and given the challenge of rural medicine, this can introduce several barriers and potentially affect outcomes of patients. As health care has shifted to large tertiary care centers integrating with community practice, the findings in this study are potentially hopeful demonstrating the impact of collaborative care. Although our patient cohort predated the COVID 19 pandemic, improvement in telehealth during the pandemic may help to provide specialized care without the burden of travel. Clinical trial enrollment and some follow up visits may be managed by remote monitoring.

This study has several limitations. First, this is a single center, retrospective study including a limited number of patients. Since next generation sequencing was introduced at the cancer center in 2015 and became more integrated in 2018, most patients in this study had follow up of less than 2 years. Patients have not been followed long enough to have sufficient survival data. Another limitation is that the majority of patients included in this study were non‐Hispanic white. Although there was not sufficient representation of other races to identify a survival difference, the study allowed for comparison of white patients from higher socioeconomic status to those from a lower socioeconomic status. Another limitation is that patients were stratified by current area of residency. Information regarding the length of residency in this area was not collected. It is possible that a patient could have moved into an urban or rural area from a different area prior to their AML diagnosis. In addition, socioeconomic status was not collected directly from the patients; rather it was extrapolated from the patient's zip code and may not reflect the true socioeconomic status of the patient as discussed above. More well‐resourced patients may have chosen to travel to the academic center, thus introducing bias.

While there continues to be progress in the care of cancer patients from rural areas including rural Appalachia, there are potential concerns on the horizon. One large change in health care over the past decade has been a transition from small intimate community care hospitals to large network health systems that may require high patient volumes to maintain their margin. Potential concerns with this transition include further limitations to access high quality specialty care required by patients with cancer who live in these rural communities.

Our center has a commitment to community outreach and engagement, and to serve rural Appalachia and other rural areas in our state. Outreach and engagement activities that will have short‐ or long‐term impact are informed by the catchment area through formal and informal interactions with regional community groups. There is extensive collaboration including community action teams to focus on cancer screening, tobacco reduction, and healthy behaviors. Our Cancer Center Without Walls represents our commitment and efforts to reach and collaborate with our catchment area, shares information, provides access to research and tertiary care. In addition, we participate in many statewide initiatives such as the Virginia Rural Health Association to improve care in rural areas.

In conclusion, this study evaluated the patient characteristics, genetics, and survival outcomes of AML patients in a single academic center treated in Virginia. The initial hypothesis was that rural patients would have worse cytogenetics, mutations, and OS; however, there were no differences observed between patients from an urban or rural area. In addition, patients from urban and rural areas, despite differences in poverty level and education, had similar rates of receiving induction chemotherapy and proceeding with HCT. The similar OS rates may be attributed to many factors including intense telehealth, frequent communication with referring physicians, and patient treatment preferences. This highlights the importance of continuing to strengthen collaborative efforts between academic oncologists and local oncologists. Future studies incorporating the collection of geographic and socioeconomic data in a prospective study are planned to provide additional insight into treatment patterns and survival outcomes for patients from rural areas.

## AUTHOR CONTRIBUTIONS

**Daniel Reed:** Data curation; investigation; methodology. **Raj Desai:** Formal analysis; methodology. **Eli Williams:** Data curation; investigation; methodology. **Rajesh Balkrishnan:** Formal analysis; methodology. **Michael Keng:** Conceptualization; data curation; investigation; methodology. **Karen Ballen:** Conceptualization; data curation; investigation; methodology.

## CONFLICT OF INTEREST

Krista M. Isaac has no conflicts of interest. Daniel R. Reed has no conflicts of interest. R. P. Desai has no conflicts of interest. E. Williams has no conflicts of interest. R. Balkrishnan is a consultant for Merck and Company. M. K. Keng is on the advisory committee for Agios. K. K. Ballen has no conflict of interest.

## ETHICS STATEMENT

This study received IRB approval (IRB 21410) from the University of Virginia Health System and given the retrospective nature of this study, patient consent was waived.

## Data Availability

The data that support the findings of this study are available on request from the corresponding author. The data are not publicly available due to privacy or ethical restrictions.
